# AFM-Based Monitoring of Enzymatic Activity of Individual Molecules of Cytochrome CYP102A1

**DOI:** 10.3390/bios15050303

**Published:** 2025-05-10

**Authors:** Yuri D. Ivanov, Natalia S. Bukharina, Ivan D. Shumov, Oleg N. Afonin, Vadim Y. Tatur, Anna V. Grudo, Alexander I. Archakov

**Affiliations:** 1 Institute of Biomedical Chemistry, 10, Pogodinskaya St., Moscow 119121, Russia; natalia.bukho@gmail.com (N.S.B.); shum230988@mail.ru (I.D.S.); sunweb@mail.ru (O.N.A.); alexander.archakov@ibmc.msk.ru (A.I.A.); 2 Foundation of Perspective Technologies and Novations, Moscow 125315, Russia; v_tatur@mail.ru; 3 Institute of Bioorganic Chemistry, National Academy of Sciences of Belarus, 220084 Minsk, Belarus; vasilevskaya.av@gmail.com

**Keywords:** cytochrome P450, atomic force microscopy, single-molecule enzymology, enzymatic activity, biosensor

## Abstract

Herein, we report the use of a nanotechnology-based approach for the study of enzyme-functionalized mica surfaces. Atomic force microscopy (AFM) has been employed for the determination of the catalytic activity of single molecules of heme-containing cytochrome P450 CYP102A1 (CYP102A1) enzyme, which was immobilized on the surface of a mica chip. Height fluctuations in individual molecules of the enzyme were measured under near-native conditions by AFM measurements in liquid using a cantilever with a 10 to 20 nm tip curvature radius. We have found that in the process of enzymatic catalysis, the mean amplitude of height fluctuations in individual enzyme molecules is 1.4-fold higher than that of enzyme molecules in an inactive state. The temperature dependence of the mean amplitude of height fluctuations in cytochrome CYP102A1 has been revealed, and the maximum of this dependence has been observed at 22 °C. The proposed nanotechnology-based approach can be employed in studies of a wide variety of enzymes, which are important for the development of novel diagnostic tests and systems for pharmaceutical analysis. The approach developed in our work will favor further miniaturization of enzyme-based biosensors and the transition from traditional sensors to nanobiosensors.

## 1. Introduction

In recent decades, the studies of single molecules of enzymes have attracted growing attention. At the same time, enzyme-based biosensor systems have found numerous applications in drug discovery [1] and in diagnostics [2]. Single-molecule studies of enzyme’s properties, including their enzymatic activity, have formed the very base of a new direction in biochemistry. This direction was called single-molecule enzymology [3]. In this connection, nanotechnology-based methods—atomic force microscopy (AFM) [4], nanowire-based [5], and nanopore-based [6,7] ones—are of use. The use of nanotechnology-based approaches to enzyme investigation favors further miniaturization of enzyme-based analytical devices, switching from traditional sensors to nanobiosensors. Nanowire- and nanoribbon-based biosensors, though providing very high detection sensitivity [5,8], do not currently allow the investigation of enzymatic reactions. On the contrary, nanopore-based sensing devices allow one to directly monitor the functioning of single-enzyme molecules, but require high concentrations of the enzyme of interest in the test solution [7].

Atomic force microscopy (AFM) is currently one of the most widely used methods of visualization and determination of the physicochemical properties of single molecules of proteins [9], including enzymes [4], nucleic acids [10], their macromolecular complexes [11,12,13], and viral particles [14] immobilized on an atomically smooth surface of a solid substrate [15,16,17,18]. For the visualization of proteins and enzymes on such a surface, one of the dynamic modes of AFM—semi-contact mode (tapping mode)—is commonly employed [15]. This allows one to minimize the impact of the AFM probe on the studied macromolecules in the process of their visualization in order to avoid protein destruction [19]. These features of AFM allow its use for the visualization of monomers and oligomers of proteins and for their identification by the height of their AFM images, as was demonstrated with the examples of putidaredoxin reductase (PdR) and cytochrome P450 102A1 [16,20]. AFM also allows one to distinguish between single protein molecules and complexes with their redox partners in complex multicomponent systems, as was demonstrated with examples of cytochrome P450-containing monooxygenase systems [15,17,21,22]. In contrast to nanopore-based sensing devices, AFM-based techniques allow one to detect the molecules of interest even at ultra-low concentrations [23].

It should be emphasized that in the present work, the tapping mode of AFM has been employed for not only the visualization, but also for the determination of the enzymatic activity of CYP102A1 enzyme, which pertains to the superfamily of heme-containing cytochromes P450. Cytochromes P450 plays a key role in xenobiotic metabolism [24,25]. Since these enzymes are key actors in drug metabolism [25], they represent important components of analytical systems for studying drug–drug interactions [1].

CYP102A1 catalyzes the monooxygenation of fatty acids [26]. CYP102A1 is a self-sufficient enzyme, and this means that no partner protein is required for its functioning [26,27]. This makes CYP102A1 a very convenient model enzyme for experimental studies of cytochromes P450, and this is why it has been selected as an object in our study, allowing us to simplify the scheme of experiment on the AFM-based determination of the cytochrome’s enzymatic activity. In solution, CYP102A1 is known to exist in the form of monomers, dimers, and higher-order oligomers [20,28,29]. Neeli et al. [29] reported the *K_d_* value for the dimerization of CYP102A1 monomers to be 1.1 ± 0.2 nM. In a number of studies [29,30,31,32,33], the enzymatic activity of CYP102A1 in the reaction of lauric acid hydroxylation (in (ω-1), (ω-2), and (ω-3) positions [34]) in solution was investigated; the turnover rate (*k_cat_*) values reported in these studies differ significantly: *k_cat_* = 53 s^−1^ [29], 46 s^−1^ [30], 86 s^−1^ [31], 26 s^−1^ [32], and 2 s^−1^ [33].

Our study is aimed at the elaboration and enhancement of the AFM-based method of determination of individual molecules of cytochromes pertaining to the P450 superfamily. In the experiments reported herein, we have measured the enzymatic activity of CYP102A1 by AFM, directly monitoring the behavior of individual molecules of the enzyme. This is in contrast to the approach employed in the above-mentioned studies [29,30,31,32,33], in which enzymatic activity values were determined by spectroscopic or isotopic (radioactive labeling) methods. In the latter method, the signal is received from a large ensemble of enzyme molecules, and the activity value is determined in such a way that it is, thus, averaged over this molecular ensemble. In this respect, the AFM-based determination of the activity of individual molecules is fundamentally different.

The AFM-based determination of CYP102A1 enzymatic activity is based on the monitoring of an increase in the amplitude of the enzyme molecules’ height fluctuations in the process of lauric acid hydroxylation. This AFM-based monitoring of CYP102A1 height value throughout the catalytic process has allowed us to register a 1.4-fold increase in the mean amplitude of CYP102A1 height fluctuations upon the enzyme functioning. The catalytic cycle time is the mean value of time between events of maximum excitation of the CYP102A1 globule throughout its functioning during the observation period. The temperature dependence of CYP102A1 globule excitation in the process of enzyme functioning has been determined.

The nanotechnology-based approach proposed herein can be of use in the development of novel enzyme-based nanoscale biosensors. These enzyme-based biosensors can find their application in diagnostics and in drug discovery.

## 2. Materials and Methods

### 2.1. Reagents and Enzyme

A total of 10 mM phosphate-buffered saline (Dulbecco’s modified; PBSD) with a pH of 7.4, containing 8 mM sodium phosphate, 2 mM potassium phosphate, 140 mM sodium chloride, and 10 mM potassium chloride was purchased from Pierce (USA). The sodium salt of lauric acid, NADPH, and 1-phenylimidazole were purchased from Sigma (St. Louis, MO, USA). Hydroxylauric acid standard (of 99.0% purity) was purchased from SynFine Research, Inc. (Richmond Hill, ON, Canada). All the aqueous solutions were prepared with ultrapure deionized water, which was purified with a Simplicity UV system (Millipore, Molsheim, France).

Wild-type cytochrome CYP102A1 was kindly donated by Professor Dr. A.W. Munro; the enzyme was expressed and prepared as described elsewhere [29]. The enzyme was also expressed by Dr. A.V. Grudo. The absorbance spectra of CYP102A1 were obtained with an Agilent Model 8453 diode array spectrophotometer (Agilent Deutschland GmbH, Waldbronn, Germany) at 25 °C. The concentration of purified CYP102A1 was determined according to the technique described by Omura and Sato [34] based on the differential absorbance spectrum of the carboxy complex of the enzyme’s reduced form, given that the extinction coefficient for the difference in absorbance at 450 and 490 nm was equal to 91 mM^−1^cm^−1^.

### 2.2. Preparation of Samples for AFM Measurements

CYP102A1 enzyme was non-covalently immobilized onto freshly cleaved mica substrates by direct surface adsorption (SPI, West Chester, PA, USA). With this purpose, a 2 µL volume of 0.5 µM CYP102A1 solution in 10 mM PBSD buffer was dispensed onto a piece of freshly cleaved mica (SPI, USA). At that, the temperature of the enzyme solution was 4 to 10 °C. The enzyme solution was incubated on mica for three minutes and then washed away with deionized water. The so-treated substrate was placed into an AFM liquid cell filled with pure 2.5 mM PBSD. Such a low salt concentration in the buffer was used in order to avoid the influence of salt deposits on the substrate surface on the quality of AFM images [35].

In order to determine whether or not there is adsorption of non-specific objects on mica, the control blank experiments were also performed at the enzyme immobilization step. Namely, a 2 µL volume of 10 mM protein-free PBSD buffer was dispensed onto control mica, and incubated thereon instead of CYP102A1 enzyme solution for three minutes.

### 2.3. AFM-Based Monitoring of Enzymatic Activity of Individual CYP102A1 Molecules

The AFM measurements were performed in the tapping mode in liquid with a Dimension 3100 atomic force microscope (Bruker, Billerica, MA, USA) equipped with DNP-S10 AFM probes (Bruker, USA; force constant 0.32 to 0.58 N/m; nominal curvature radius 10 to 20 nm). The purity of all the solutions was checked by AFM prior to their use in the AFM experiments; the height of non-specific objects revealed on mica during these checks did not exceed 0.5 nm. The temperature in the AFM liquid cell was kept constant and set to 22 °C—except for experiments on the determination of the temperature dependence of enzymatic activity. In the latter case, the temperature in the cell varied between 17 and 28 °C.

The AFM-based monitoring of enzymatic activity consisted of obtaining time dependence of the height fluctuations in individual molecules of CYP102A1 in the process of the catalytic cycle of lauric acid hydroxylation reaction according to the method described elsewhere [36]. With this purpose, a preliminary scanning of the substrate surface was performed in order to select an area containing the adsorbed enzyme molecule of interest. After that, the scanning of this selected area was started. At the moment when the scanning probe reached the middle region of the selected molecule, scanning in the slow scan direction (along the *Y*-axis) was disabled. In this way, a cross-section image of the selected enzyme molecule of interest was obtained in a time-base sweep. The scanning frequency was set to the highest value technically possible for this atomic force microscope (1 to 1.3 Hz).

The AFM-based monitoring of the enzymatic activity was performed in four sequential steps. Firstly, the measurements were performed in pure 2.5 mM PBSD with pH 7.4 (step 1). Secondly, sodium laurate (substrate) solution in buffer was added into the liquid cell to the final substrate concentration of 500 µM (step 2). Thirdly, the buffered solution of NADPH electron donor was added into the cell to its final concentration of 200 µM (step 3). In the fourth step, the catalytic reaction was stopped by the addition of 1-phenylimidazole inhibitor to its final concentration in the cell of 5 mM.

At all four steps, cross-section images of no less than ten different molecules on the substrate were obtained as described above. Observations upon the addition of the substrate, the electron donor, or the inhibitor were performed with a delay from the moment of addition of either of the enzymatic reaction components *t_lag_*~5 min. This time was required to stabilize the AFM scanning conditions after the hydrodynamic disturbance in the liquid in the cell, which occurred upon the addition of the solution with either of the reaction components.

### 2.4. AFM Data Processing

The processing of the AFM data and measurements of heights of the AFM images of the visualized molecules were performed with the «AFMdtpr» software v. 1.0 (developed in IBMC).

The heights of CYP102A1 molecules were determined as heights of the respective maxima of *ρ*(*h*) distributions (density functions [37]) of their AFM images by height, which were calculated as follows:

*ρ*(*h*) = *N_h_*/*N* × 100% (1)

where *N_h_* is the number of molecules of height *h*, and *N* is the total number of visualized molecules.

Approximation of the experimental dependence (1) was performed using the following Gaussian function [20]:

(2) ρh=∑ρih=∑i=12Aexp(−4ln⁡2h−hc2w−2)wπ4ln⁡(2)

where *A*, *h_c_*, and *w* are the variable approximation parameters. In this case, the position of the *ρ*(*h*) maximum was determined as the maximum of the approximating Function (2) for each distribution. The analysis of the approximation of experimentally obtained *ρ*(*h*) distributions was based on the χ^2^ criterion. The experimental curve was approximated by the sum of two exponentials, and the AFM images of the visualized molecules were divided into two groups with respective height maxima (*h_max1_* and *h_max2_*).

### 2.5. Processing of the Cross-Section Images of CYP102A1 Molecules

After the analysis of the AFM images of cross-sections of individual CYP102A1 molecules, the values of the amplitude of their fluctuations were averaged. Processing of the cross-section images was performed with the homemade «RMS-IBMC» software ver. 1 (developed in IBMC). This software allowed us to obtain a trajectory of fluctuations (*h_max_*(*t*) trace) of an enzyme molecule based on the image of its cross-section. The *h_max_*(*t*) trace represented the time dependence of the molecule’s height maxima. Based on this trace, the RMS_CYP102A1_ (root mean square) value was calculated. Analogous *h*(*t*) traces were obtained for several areas (three to four in each frame) of the mica substrate surface adjacent to the investigated enzyme molecule in order to determine the background noise level upon the measurements on mica.

Upon opening the AFM image to be processed with the software, the scan frequency (in Hz) and the number of pixels along the image’s *X*-axis should be indicated in the software settings. Subsequently, the following ranges (the pixel numbers) should be specified: the range along the *X*-axis, within which the maximum height value is determined; and the range along the *Y*-axis, which specifies the length of the trajectory. The selected ranges can be moved in the image with scroll bars. The *h_max_*(*t*) trace, determined for each investigated enzyme molecule in this way, was saved as a text file for further mathematical treatment.

In order to compare the trajectories of different molecules, linear approximation was performed for each of them. After that, it is convenient to present Δ*h*(*t*) in graphic form as a cross-section image by the X-axis. Based on the Δ*h*(*t*) trace, the amplitude *A*_CYP102A1_ of height fluctuations during the observation period Δ*t* was calculated for an individual CYP102A1 molecule as follows:

(3) $$A_{BM3}=\sqrt{\frac{1}{n}\sum_{i=1}^{n}{\left(∆h_{i}\right)}^{2}}$$

where Δ*h_i_* is the height fluctuation in the time point *i*, and *n* is the number of points in the trace. The Δ*t* value was equal to a time period averaged over the entire set of recorded traces, providing a convenient comparison of fluctuation amplitudes of different molecules. This Δ*t* value ranged from 15 to 30 s.

The mean value of the fluctuation amplitude of CYP102A1 molecules *Ā*_CYP102A1_ was estimated by averaging the *A*_CYP102A1_ values obtained for individual molecules of the enzyme. The *Ā*_CYP102A1_ values at each step of the catalytic cycle of the enzyme were obtained by averaging *A*_CYP102A1_ for at least ten different molecules. The differential value Δ*Ā* for the mean values of fluctuation amplitude of the active enzyme (*Ā*_CYP102A1_^active^, 3rd step of the catalytic cycle) and the resting enzyme (*Ā*_CYP102A1_^resting^, 2nd step of the catalytic cycle) was estimated as follows:

Δ*Ā_CYP_*_102*A*1_ = *Ā_CYP_*_102*A*1_*^active^* − *Ā*_CYP102A1_^resting^ (4)

The differential value for an individual molecule Δ*A_CYP102A1_* was estimated analogously:

Δ*A*_*CYP*102*A*1_ = *A*_*CYP*102*A*1_*^active^* − *Ā*_CYP102A1_^resting^ (5)

### 2.6. Steady-State CYP102A1 Activity Study by Spectrophotometry

In order to determine the background noise level, a signal from the area of the AFM substrate surface without adsorbed enzyme was registered at each step of AFM measurements. The traces of fluctuation of the mica surface in the cross-section AFM images were indicated as *h_mica_*(*t*). Thus, the value of fluctuations in the mica surface *A*_mica_ was determined in each frame. These values were subsequently averaged (*Ā*_mica_) for a given step of the catalytic cycle.

The measurement error *σ* for the mean values of height fluctuation amplitude was estimated based on a 95% confidence probability:

(6) $$\sigma =\pm t\sqrt{\sum_{i}\Delta_{i}^{2}/(n\left(n-1\right))}$$

where Δ*_i_* is the deviation of the result of each experiment from the mean value, *n* is the number of experiments in the series, and *t* is Student’s coefficient.

In order to confirm the enzymatic activity of CYP102A1 in the reaction of lauric acid hydroxylation in the presence of NADPH, the spectrophotometry-based technique described by Neeli et al. [29] was employed. The spectrophotometry measurements were performed with an Agilent 8453 spectrophotometer (Agilent Deutschland GmbH, Waldbronn, Germany) in a quartz cell with a pathlength of 1 cm (Agilent Deutschland GmbH, Waldbronn, Germany) in 50 mM potassium phosphate buffer at pH 7.0. The final concentrations of the enzyme, its substrate (sodium laurate), and the electron donor NADPH in the cell were 40 nM, 500 µM, and 200 µM, respectively. A 1.5 mL volume of the initial 8 × 10^−5^ M enzyme solution was added to the buffer in the cell containing the subsrate, and stirred thoroughly. After that, a 30 µL volume of 14.9 mg/mL NADPH solution was pipetted into the cell upon stirring, so that the final volume of the solution in the cell was 3 mL. The measurement was started immediately after the addition of NADPH. The decrease in absorbance of the solution in the cell at 340 nm (Δ*A_340_*) was monitored for ten minutes, and the resulting time dependence Δ*A_340_*(*t*) was recorded.

## 3. Results

### 3.1. AFM Visualization of CYP102A1

**Figure 1:**
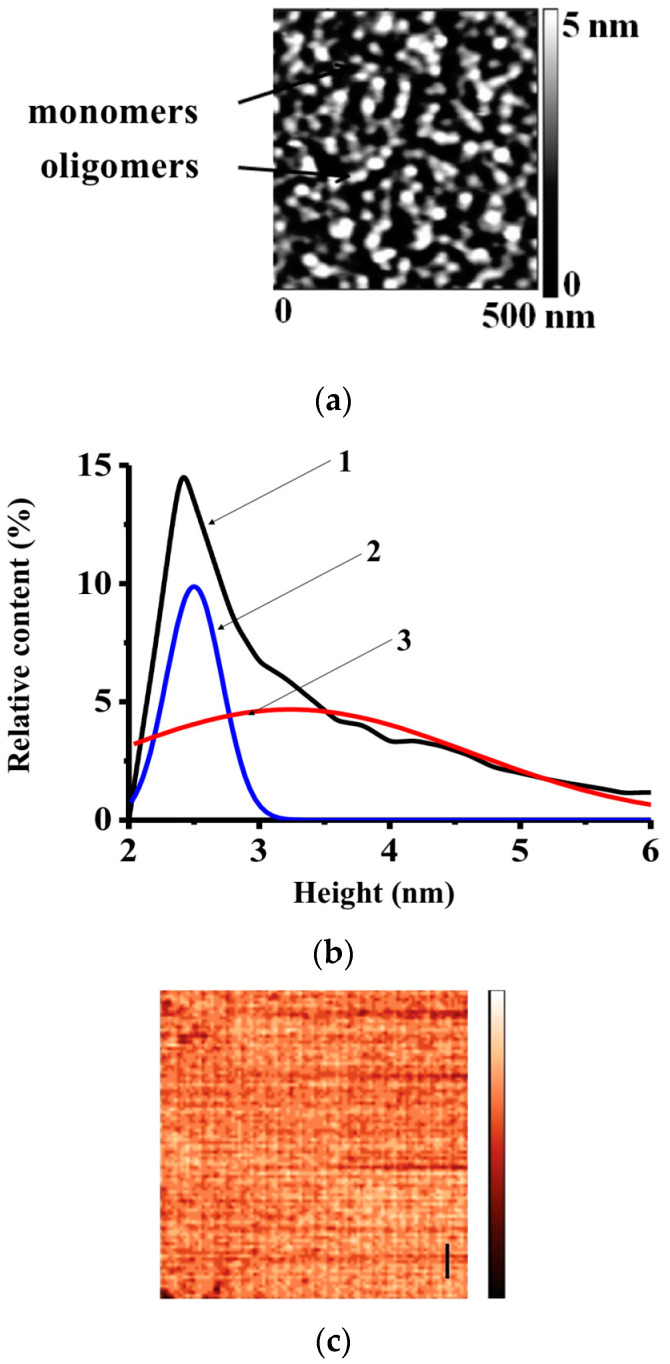
Typical AFM images of the monomers and oligomers of CYP102A1 adsorbed on mica (**a**), and *ρ*(*h*) distributions (density functions) of their AFM images by height (**b**). The image in panel (**c**) (scale bar, 200 nm; Z scale, 1 nm) was obtained in the control blank experiment with the use of protein-free 10 mM PBSD buffer instead of enzyme solution. A total of 0.5 mM CYP102A1 in 10 mM PBSD buffer with pH 7.4 was dispensed onto a piece of mica, which was then washed with pure water and placed into 2.5 mM PBSD. The measurements were performed by AFM in the tapping mode in liquid at 22 °C. Curve (1) shows the experimental *ρ*(*h*) distribution obtained from the AFM data, while curves (2) and (3) show the calculated distributions for the monomers and oligomers of the enzyme, respectively.

At the first step, CYP102A1 was visualized by AFM for further study of their enzymatic activity. As was noted in the Introduction Section, AFM allows one to visualize single enzyme molecules and to measure their height. These capabilities of AFM were employed for the visualization of the monomers and oligomers of CYP102A1, their identification by height, and the determination of the degree of CYP102A1 oligomerization, as was demonstrated earlier [20]. The distribution of the AFM images of CYP102A1 molecules, immobilized on mica, by height shown in Figure 1b indicates the presence of objects with maximum height at 2.7 ± 0.1 nm, which were attributed to the monomeric form of the enzyme, while objects with maximum height at 3.5 ± 0.3 nm were attributed to its oligomeric form. At that, the calculation of the number of the monomers and the oligomers showed that the ratio between the monomeric and the oligomeric form of the enzyme was 0.5:0.5 (Figure 1).

The characteristic AFM image displaying the topography of the mica substrate used in control blank experiments is shown in Figure 1c. This image illustrates the absence of any objects non-specifically adsorbed from the working buffer, thus indicating the absence of undesired influence of non-specific objects on the experiments with the enzyme.

### 3.2. Determination of CYP102A1 Enzymatic Activity by AFM

The enzymatic activity of individual CYP102A1 molecules was performed by monitoring fluctuations in their height by AFM in the process of the enzymatic catalysis of lauric acid hydroxylation in the presence of NADPH electron donor. The amplitude of CYP102A1 molecules’ height fluctuations were measured in 2.5 mM PBSD buffer under the following conditions:

(A) In pure buffer (the first step of the catalytic cycle);
(B) In buffer in the presence of 500 µM sodium laurate (the substrate; the second step of the catalytic cycle);
(C) In buffer in the presence of 500 µM sodium laurate (the substrate) and 200 µM NADPH (the electron donor); under these conditions, the reaction of laurate hydroxylation is performed (third step of the catalytic cycle);
(D) In buffer in the presence of 5 µM 1-phenylimidazole (the inhibitor); the inhibitor was added in order to terminate the enzymatic reaction of substrate hydroxylation (fourth step of the catalytic cycle).

**Figure 2:**
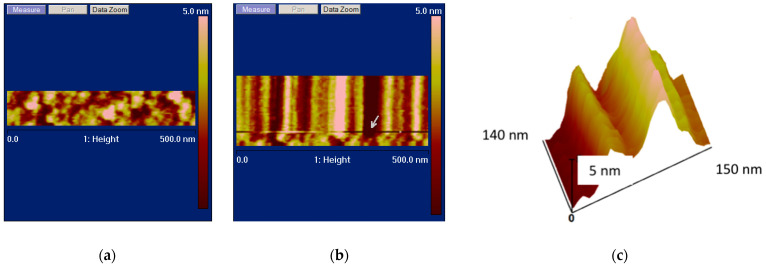
Typical AFM images of CYP102A1 adsorbed on mica. The images of the enzyme molecules in resting state were obtained in liquid in the standard panoramic AFM scanning mode (**a**), and in the mode of cross-section scanning with disabled scanning along the *Y*-axis (**b**). In panel (**b**), the white arrow indicates the moment of disabling the scanning along the *Y*-axis. Panel (**c**) displays a 3D AFM image of the cross-section of enzyme molecules (obtained from the image shown in panel (**b**)), illustrating the time-base sweep of the molecules’ topography cross-section. Experimental conditions: AFM scanning—2.5 mM PBSD buffer in the presence of 500 µM sodium laurate (the substrate; second step of the catalytic cycle).

**Figure 3:**
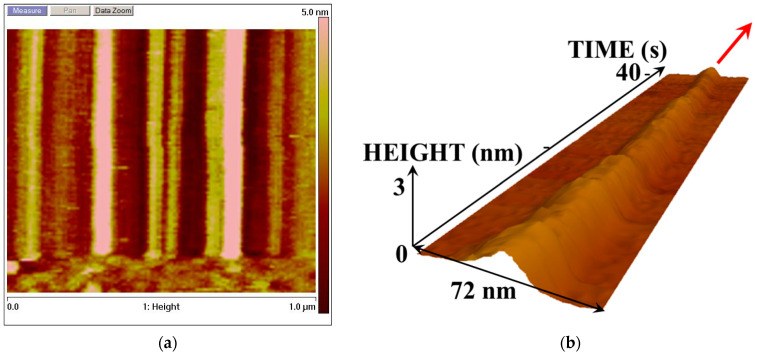
Typical AFM images of CYP102A1 adsorbed on mica. The images of the enzyme molecules under the conditions of catalytic reaction were obtained in liquid. Panel (**a**) displays switching from the standard panoramic AFM scanning mode to the mode of cross-section scanning with disabled scanning along the *Y*-axis. Panel (**b**) displays a 3D AFM image of the cross-section of a single enzyme molecule, illustrating the time-base sweep of its topography cross-section. Experimental conditions: 2.5 mM PBSD buffer in the presence of 500 µM sodium laurate and 200 µM NADPH (third step of the catalytic cycle).

It should be emphasized that the height of the majority of the BM3 molecules exceeds 2.5 nm. Using these images, it is quite difficult to distinguish 0.5 Å fluctuations in these molecules. In order to monitor the enzyme molecule’s behavior during its functioning, the tip of the AFM probe should be precisely positioned over the top of this molecule and track its fluctuations during its catalytic cycle. It is difficult to analyze this based on conventional panoramic AFM images. Thus, the time traces of height fluctuations in the molecule’s top should be recorded while scanning in the slow scan direction (along the *Y*-axis) is disabled. And this is what we have performed in our reported experiments. Figure 2 and Figure 3 display the AFM images reflecting the dynamics of changes in the enzyme molecule in its cross-section. These images were obtained when scanning along the *Y*-axis was disabled, and the AFM probe tip was only scanned in one direction.

In Figure 2, one can distinguish the higher and lower AFM images of the enzyme molecules in the resting state: the higher (>3 nm) images are attributed to the enzyme oligomers, while the lower (<2.5 nm) ones pertain to the monomeric form of the enzyme. Both forms of the enzyme are illustrated by the 3D image in Figure 2c. Slight changes in the images of the enzyme molecules over time are caused by thermal drift.

Figure 3 displays the AFM images reflecting changes in the height of the CYP102A1 molecules under the conditions of the catalytic reaction (on the third step of the catalytic cycle).

**Figure 4:**
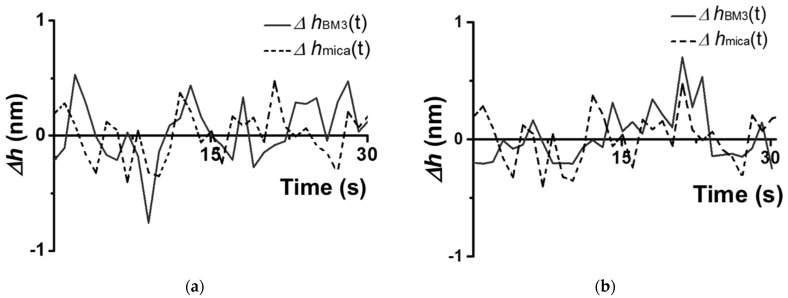
Example of characteristic traces of height fluctuations obtained upon the AFM-based monitoring of enzymatic activity of a CYP102A1 molecule in pure 2.5 mM PBSD buffer (**a**), and in the presence of 500 µM sodium laurate (**b**). The traces Δ*h _CYP102A1_*(*t*) obtained for the enzyme molecule are shown with a solid line, while the traces Δ*h_mica_*(*t*) obtained for the mica surface are shown with a dashed line. The height of the enzyme molecule was 3.8 ± 0.1 nm in both cases.

**Figure 5:**
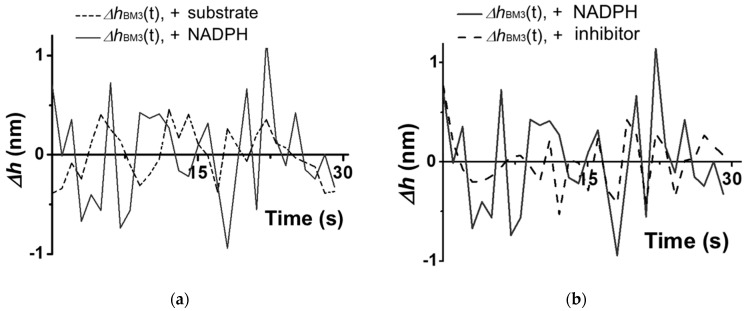
Example of characteristic traces of height fluctuations obtained upon the AFM-based monitoring of enzymatic activity of a CYP102A1 molecule at the 2nd and 3rd (**a**), and at the 3rd and 4th (**b**) steps of the catalytic cycle. In panel (**a**), the traces Δ*h* _CYP102A1_(*t*) obtained for the CYP102A1 molecule in the presence of sodium laurate before the addition of NADPH are shown with a dashed line, while the traces Δ*h* _CYP102A1_(*t*) obtained for this molecule in the presence of sodium laurate after NADPH addition are shown with a solid line. In panel (**b**), the traces Δ*h* _CYP102A1_(*t*) obtained for the enzyme molecule in the presence of sodium laurate and NADPH before the addition of 1-phenylimidazole are shown with a solid line, while the traces Δ*h* _CYP102A1_(*t*) obtained in the presence of 1-phenylimidazole are shown with a dashed line. The height of the enzyme molecule was 3.8 ± 0.1 nm in all cases.

Characteristic traces observed for a CYP102A1 molecule under the conditions (A–D) are shown in Figure 4 and Figure 5.

The traces shown in Figure 4 and Figure 5 were obtained based on the cross-section images of CYP102A1. These traces indicate that the presence of the substrate (sodium laurate) does not change the enzyme molecule’s fluctuations (Figure 4b)). At the same time, the addition of the NADPH electron donor leads to a considerable increase in the amplitude of the fluctuations (Figure 5a). The values of this amplitude become comparable to its initial values after the addition of the 1-phenylimidazole inhibitor (Figure 5b).

Once again, the amplitude of CYP102A1 height fluctuations did not change at the step of the addition of the sodium laurate (the substrate). In contrast, the addition of NADPH induces the catalytic reaction, leading to an increase in the amplitude of the fluctuations. Namely, the values of these fluctuations become higher than those observed before the NADPH addition. These fluctuations reflect stochastic events of increase–decrease in the amplitude. That is, in the process of catalysis, the amplitude of fluctuations in individual enzyme oligomers becomes higher than that observed before the NADPH addition. The amplitude of the fluctuations decreases after the addition of the inhibitor. Thus, in the process of catalysis, occurring upon the participation of CYP102A1 oligomers, we observed an increase in the amplitude of fluctuations of its height with characteristic intervals between them. This means that the catalytic activity of the enzyme can be characterized by two parameters—by a dynamic parameter and by a time parameter. Namely, the amplitude of the enzyme’s height fluctuations represents the dynamic parameter, while the time between the events of fluctuations with increased amplitude represents the time parameter.

It is interesting to analyze the characteristics of these fluctuations averaged over several enzyme molecules. To this aim, averaging for CYP102A1 oligomers was performed over ~30 molecules. The resulting data are listed in Table 1.

The data listed in Table 1 indicate that the amplitude of height fluctuations differed under different conditions. Namely, while the presence of the sodium laurate substrate had no influence on the amplitude and the σ value at the first and the second steps amounted to Δ*Ā* = 0.25 ± 0.03 nm (with account taken for the noise from mica), an increase in the amplitude to the values of Δ*Āactive* = 0.36 ± 0.03 nm was observed under the conditions of hydroxylation reaction after the addition of NADPH electron donor at the third step. That is, the mean value of the amplitude of height fluctuations in individual CYP102A1 enzyme molecules in the process of catalysis is 1.4-fold higher than that in the resting state. After the subsequent addition of 1-phenylimidaazole inhibitor to the reaction medium (4th step), the amplitude of the fluctuations decreased to its initial values Δ*Ā* = 0.21 ± 0.03 nm. At that, upon averaging over a number of molecules, the amplitude of fluctuations in the active molecule exceeds that of the inactive one.

Thus, upon the addition of NADPH electron donor to the reaction medium (i.e., in the process of hydroxylation reaction) the differential value of the amplitude of height fluctuations was Δ*Ā* _CYP102A1_ *= Ā* _CYP102A1_^active^ − *Ā* _CYP102A1_^resting^ ≈ (1.1 ± 0.4) Å. Accordingly, this value can be used as a measure of the activity of individual CYP102A1 molecules.

The enzymatic activity of CYP102A1 is known to depend on temperature [38,39]. Importantly, the temperature dependence of the enzymatic activity of CYP102A1 immobilized in gel has an arched shape with a maximum at 25 °C [38].

**Figure 6:**
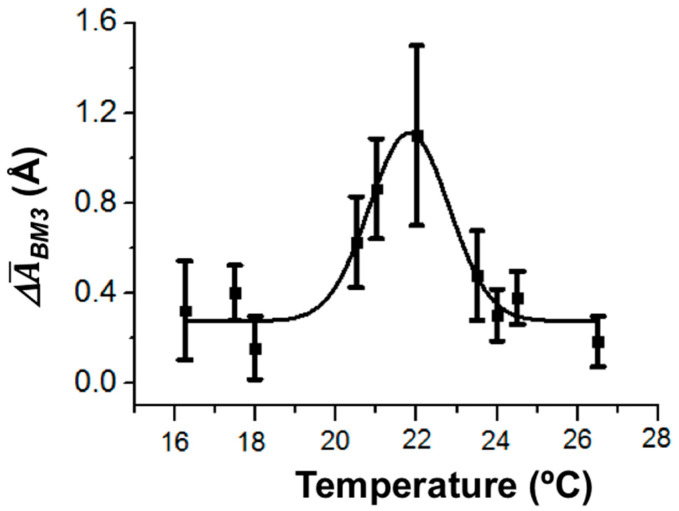
Temperature dependence of CYP102A1 height fluctuations. Experimental conditions: 0.5 mM CYP102A1 in 10 mM PBSD buffer with pH 7.4 was dispensed onto a piece of mica, which was then washed with pure water and placed into 2.5 mM PBSD. The measurements were performed by AFM in the tapping mode in liquid.

In our study, the temperature dependence of the amplitude of height fluctuations in the enzyme molecule as a dynamic parameter of its enzymatic activity has also been determined. The values of the amplitude were averaged over ~20 enzyme molecules. The maximum of this temperature dependence was observed at 22 °C (Figure 6).

Thus, in our experiments, the temperature dependence of the amplitude of CYP102A1 height fluctuations (the dynamic parameter) was observed. This is in coincidence with the known fact of the temperature dependence of the CYP102A1 enzymatic activity.

### 3.3. Determination of CYP102A1 Enzymatic Activity by a Standard Spectrophotometric Assay

**Figure 7:**
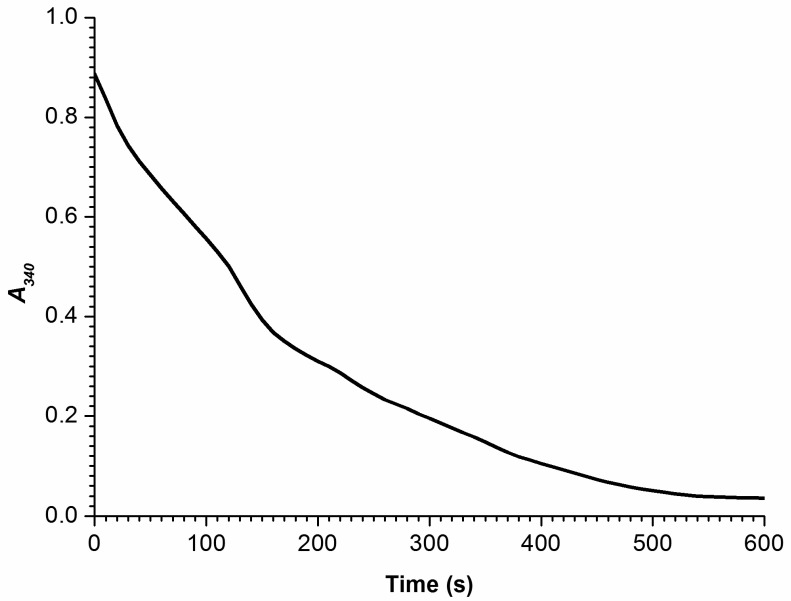
Time dependence of a decrease in absorbance Δ*A_340_* of the reaction solution at 340 nm. Experimental conditions: the concentrations of the enzyme, the substrate (sodium laurate), and the NADPH electron donor were 4 × 10^−8^ M (40 nM), 500 µM, and 200 µM, respectively; 50 mM potassium phosphate buffer (pH 7.0); optical path length 1 cm; temperature 25 °C.

The enzymatic activity of CYP102A1 in the reaction of lauric acid hydroxylation in the presence of NADPH electron donor was determined by spectrophotometry using a standard assay reported by Neeli et al. [29] as described in Section 2. The time dependence of absorbance of the reaction solution, containing 500 µM sodium laurate and 200 µM NADPH in 50 mM potassium phosphate buffer, at 340 nm has been recorded. This Δ*A_340_(t)* dependence is shown in Figure 7.

The Δ*A_340_(t)* curve shown in Figure 7 indicates NADPH oxidation, and illustrates that during the first 40 s of observation, the specific reaction rate at 4 × 10^−8^ M (40 nM) enzyme concentration was about *k_cat int_*=20 ± 1 s^−1^.

## 4. Discussion

In order to describe enzyme kinetics, the Michaelis–Menten theory is commonly used. Within the framework of this theory, the behavior of a large ensemble of enzyme molecules is considered. As mentioned in Section 1, the enzymatic activity of CYP102A1 was previously measured in several studies [29,30,31,32,33]. At the same time, the methods of enzymatic activity determination were described in detail by only Neeli [29] and Peterson [33]. So, Neeli et al. [29] determined the enzymatic activity of CYP102A1 by the steady-state kinetic measurement of the characteristic rate of specific oxidation of NADPH at 25 °C and pH 7.0 in 50 mM potassium phosphate buffer. The specific rate of NADPH oxidation was relatively constant at enzyme concentrations ≥ 10 nM, amounting to 50 s^−1^. In our experiments, the specific rate of NADPH oxidation was of the same order of magnitude, making up about *k_cat int_* = 20 s^−1^ at 40 nM enzyme concentration. These measurements, however, give an overestimated value of enzymatic activity, since the latter was calculated from the integral values of NADPH oxidation rate.

In the study reported herein, we propose a fundamentally different approach, which consists of the analysis of the activity of an individual enzyme molecule—not the molecular ensemble. This approach has allowed us to directly monitor the activity of an individual enzyme molecule during the period of observation. For signal processing, we propose to use a value of an increase in the signal with respect to the cut-off level. The latter represents the level of AFM-registered amplitude of height fluctuations in CYP102A1 in its resting state. This level is determined in a liquid medium in the presence of the substrate but in the absence of the NADPH electron donor. As mentioned in Section 3, an increase in the amplitude of fluctuations in CYP102A1 molecule height is observed when the enzyme is in its active state. Namely, the averaged value of these fluctuations is 1.4-fold higher than that of inactive enzyme molecules. Thus, the amplitude of molecule height fluctuations, indeed, represents a dynamic parameter of the enzymatic activity. The increase in the mean amplitude of fluctuations in individual molecules in the active state—in comparison with their resting state—is caused by a change in the molecule conformation during catalysis. In the process of catalysis, the enzyme molecule changes its spatial conformation, and this transition from one conformation to another is accompanied by an increase in the molecule’s height. Previously, Radmacher et al. reported an increase in the height of enzyme molecules in the process of catalysis, demonstrating this with examples of lysozyme ref. [36] and chitosanase [40].

It is interesting to compare the values of τ_cat_ obtained in our study with the data reported in the literature. Based on our data on the NADPH oxidation kinetics, τ_cat int_ = 1/(20 s^−1^) = 50 ms.

The reaction rate constant determined by spectrophotometry can be presented as

(7) $$k_{cat\;int}=\sum_{i}^{m}{\left[<k_{on}>\right]}_{i}B_{i}$$

where *m* is the number of NADPH molecules participating in the catalytic reaction, [*<k_on_>*]*_i_* is the rate constant of oxidation of *i*th molecule of NADPH, and *B_i_* is the constant for this *i*th molecule.

The value of τ_cat_ obtained based on the rate of NADPH oxidation is underestimated since it was determined by the spectrophotometric monitoring of NADPH consumption during the catalytic reaction.

**Figure 8:**
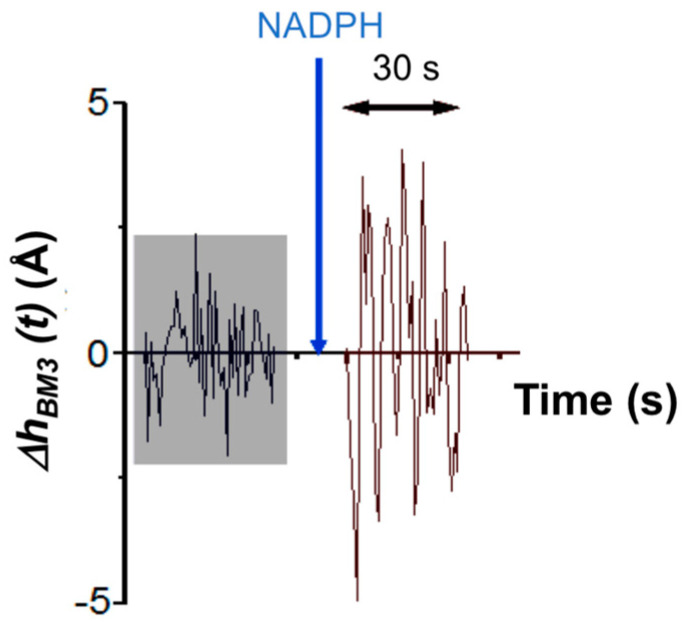
Height fluctuation traces obtained by AFM for an individual CYP102A1 molecule obtained at the 2nd and 3rd steps of the catalytic cycle. The gray block indicates the height fluctuations in the molecule in the resting state. The blue arrow indicates the time point of NADPH addition.

In the case of the AFM-based approach, τ_catAFM_ ≈ 7 s. This value is determined by analyzing the amplitude of height fluctuations in an individual CYP102A1 molecule as an integral value of the observation time period divided by the number of molecule fluctuations with an amplitude, which exceeds the value of the background noise measured during this time period. This time dependence of height fluctuations in an individual CYP102A1 molecule is shown in Figure 8.

In general, the rate constant of laurate hydroxylation by a given individual CYP102A1 molecule determined by AFM can be presented as follows:

1/τ_cat AFM_ = *k_cat AFM_* = *ᶘk_on_*(*t*) *f*(*t*) *dt*, (8)

where *k_on_*(*t*) is the true rate constant of laurate hydroxylation, and *f*(*t*) is the instrument function.

The value of τ_cat AFM_ exceeds that of τ_cat int_ (which is determined by spectrophotometry) by two orders of magnitude. This can be explained by a somewhat lower activity of mica-immobilized CYP102A1 and by the additional influence of effects connected with the re-organization of the aqueous microenvironment, which can occur in the process of the enzyme functioning. This can result in a transformation of the enzyme globule’s structure in the process of catalysis and is reflected in the form of the instrument function contribution in Equation (8). Water is known to be not isotropic: it is structured, as was shown in [41]. At that, micro-clusters exist. They have characteristic sizes of 0.5 to 50 µm [42]. The formation/decay times of these clusters amount to seconds. These time values are within the range, in which the activity of the enzyme is registered.

Thus, one can assume that the fluctuations of active CYP102A1 molecules observed by AFM can be explained by the fluctuations in the enzyme globules in the process of catalysis by the interference of these fluctuations within the enzyme molecule, as well as by the interference of the fluctuations resulting from the interaction with water clusters, which are re-organizing upon the enzyme functioning.

Our results reported herein emphasize the potential of AFM as a promising tool for single-molecule studies of enzyme functioning. In this regard, nevertheless, one should be aware of certain limitations of the AFM-based approach. These limitations are connected with the mandatory requirement to attach the enzyme of interest to a solid surface, which is subsequently scanned with the AFM probe. Such an attachment can be achieved either by non-covalent immobilization via the physical adsorption of the enzyme [22,40], or by covalent immobilization with the use of various cross-linking agents [23]. On the one hand, the attachment should be strong enough in order to prevent the enzyme’s displacement on the surface throughout the scanning; such a displacement can occur due to thermal drift or under the action of the AFM probe [36]. On the other hand, the enzyme immobilization technique should be selected very carefully, since the immobilized enzyme should retain its functional activity [43]. In our experiments, the enzyme was immobilized non-covalently, and careful thermal stabilization of the scanned sample was performed in order to minimize the thermal drift. In view of these limitations, careful selection of the experimental conditions should be performed in each particular case.

## 5. Conclusions

The functional activity of individual molecules of the CYP102A1 enzyme has been monitored by AFM. The time dependencies of height fluctuations in CYP102A1 were averaged over 30 enzyme molecules. It has been demonstrated that the mean amplitude of height fluctuations in individual CYP102A1 oligomers in the active state is 1.4-fold higher than that in the inactive (resting) state. The temperature dependence of height fluctuations in individual CYP102A1 enzyme oligomers has also been obtained. This dependence had a maximum at 22 °C.

## Figures and Tables

**Table 1:** Mean values of the amplitude of height fluctuations in individual CYP102A1 molecules *Ā_CYP102A1_* and mean values of noise observed on mica *Ā_mica_* at different steps of the CYP102A1 catalytic cycle.

Step of the Catalytic Cycle	*Ā*_CYP102A1_ ± σ, nm	*Ā*_mica_ ± σ, nm
1st step (pure buffer)	0.25 ± 0.03	0.24 ± 0.03
2nd step (500 µM sodium laurate added)	0.25 ± 0.03	0.25 ± 0.03
3rd step (500 µM sodium laurate, 200 µM NADPH added)	0.36 ± 0.02	0.23 ± 0.03
4th step (5 mM 1-phenylimidazole added)	0.21 ± 0.02	0.23 ± 0.03

## Data Availability

Data are contained within the article.

## Abbreviations

The following abbreviations are used in this manuscript:

AFM	atomic force microscopy
NADPH	Nicotinamide adenine dinucleotide phosphate
